# Comprehensive vs. standard remote monitoring of cardiac resynchronization devices in heart failure patients: results of the ECOST-CRT study

**DOI:** 10.1093/europace/euae233

**Published:** 2024-10-14

**Authors:** Cédric Klein, Claude Kouakam, Arnaud Lazarus, Pascal de Groote, Christophe Bauters, Eloi Marijon, Frédéric Mouquet, Bruno Degand, Yves Guyomar, Jacques Mansourati, Christophe Leclercq, Laurence Guédon-Moreau, ECOST-CRT study Investigators, Laurence Guedon-Moreau, Bruno Degand, Yves Guyomar, Jacques Mansourati, Dominique Babuty, Maxime Pons, Benoit Guy-Moyat, Jean-Claude Deharo, Daniel Gras, Caroline Himbert, Christophe Leclercq, Jean-Luc Pasquie, Romain Eschalier, Halim Marzak, Michel Boursier, François Jourda, Frédéric Anselme, Hervé Gorka, Olivier Billon, Laure Champ-Rigot, Mina Ait Said, Jérôme Taieb, Marc Badoz, Julien Laborderie, Mohamed Belhameche, Sylvain Ploux, Maxime de Guillebon, Antoine Dompnier, Serge Boveda, Sophie Gomes-Ferreira, Cédric Giraudeau, Michael Peyrol, Pierre Winum, Benjamin Gal, Hugues Blangy, Olivier Le Vavasseur, Alexandre Duparc, Laura Forelle, Albin Behaghel, Renaud Fouche, Gabriel Laurent, Hassan Barake, Sylvain Reuter, Pierre Sultan, Antoine Da Costa

**Affiliations:** CHU Lille, Lille, France; CHU Lille, Lille, France; Clinique Ambroise Paré, Neuilly-sur-Seine, France; CHU Lille, Lille, France; Univ. Lille, Inserm, CHU Lille, Institut Pasteur, Lille, France; Hôpital Européen Georges Pompidou, Paris, France; Hôpital privé Le Bois, Lille, France; Centre Hospitalier Universitaire Poitiers, Poitiers, France; Centre hospitalier Saint Philibert, Lomme, France; CHU Brest, Brest, France; CHU Rennes, Rennes, France; CHU Lille, Univ.Lille, Lille, France

**Keywords:** Heart failure, Cardiac resynchronization therapy, Remote monitoring

## Abstract

**Aims:**

Integrating remote monitoring (RM) into existing healthcare practice for heart failure (HF) patients to improve clinical outcome remains challenging. The ECOST-CRT study compared the clinical outcome of a comprehensive RM scheme including a patient questionnaire capturing signs and symptoms of HF and notifications for HF specific parameters to traditional RM in patients with cardiac resynchronization therapy (CRT) devices.

**Methods and results:**

Patients were randomized 1:1 to standard daily RM (notification for technical parameters and ventricular arrhythmias; control group) or comprehensive RM (adding a monthly symptom questionnaire and notifications for biventricular pacing, premature ventricular contraction, atrial arrhythmias; active group). The primary endpoint was all-cause mortality or hospitalization for worsening HF (WHF). Six hundred fifty-two patients (70.4 ± 10.3 years, 73% men, left ventricular ejection fraction 29.1 ± 7.6%, 68% CRT-Defibrillators, 32% CRT-Pacemakers) were enrolled. The COVID-19 pandemic caused an early termination of the study, so the mean follow-up duration was 18 ± 8 months. No statistically significant difference in the primary endpoint was found between the groups [59 (18.3%) control vs. 77 (23.3%) active group; log-rank test *P* = 0.13]. Among the secondary endpoints, the MLHF questionnaire showed a larger share of patients with improvement of quality of life compared to baseline in the active group (78%) vs. control (61%; *P* = 0.03).

**Conclusion:**

The study does not support the notion that comprehensive RM, when compared to standard RM, in HF patients with CRT improves the clinical outcome of all-cause mortality or WHF hospitalizations. However, this study was underpowered due to an early termination and further trials are required.

**Registration:**

Clinical Trials.gov Identifier: NCT03012490

## Graphical Abstract

**Graphical Abstract:**
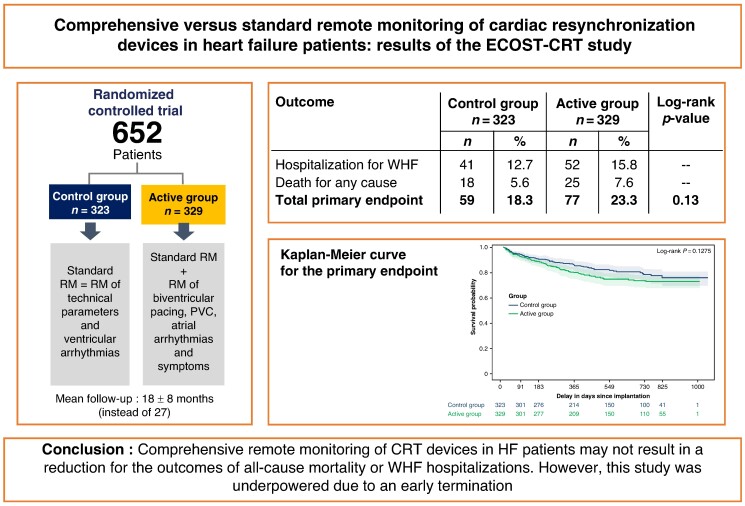
CRT, cardiac resynchronization therapy; HF, heart failure; PVC, premature ventricular contraction; RM, remote monitoring; WHF, worsening heart failure.

**What’s new?**

- The study addresses a crucial aspect of heart failure management by focusing on the remote monitoring of devices for resynchronization. For heart failure, which is a major cause of morbidity and mortality, the contribution of remote monitoring is still debated, including for resynchronized patients.
- Compared with basic cardiac resynchronization therapy defibrillator and cardiac resynchronization therapy pacemaker remote monitoring, the addition of variables including atrial fibrillation, resynchronization rate, and patient symptoms informed by a technological advance consisting of an electronic questionnaire does not seem to improve patient prognosis.

## Introduction

Remote monitoring (RM) is used in patients with chronic cardiac disease to optimize patient care. In patients with heart failure (HF) but without cardiac implantable electronic devices (CIED), RM using external devices often failed to show a positive impact on endpoints such as mortality or HF hospitalization.^1^ In contrast, RM is strongly recommended in patients with CIED, with a high level of evidence, as part of the standard of care.^2,3^ Few studies have focused on patients with HF and CIED. The IN-TIME study^4,5^ investigated patients with HF treated with implantable cardioverter-defibrillator (ICD) or cardiac resynchronization therapy defibrillator (CRT-D). Daily RM of device data such as intracardiac electrograms of ventricular and atrial tachyarrhythmia episodes, low percentage of biventricular pacing, increase in the frequency of premature ventricular contractions (PVCs), or decreased patient activity was complemented with a pre-specified scheme of interventions. This had a beneficial effect on the ‘Packer’ composite clinical score comprising all-cause death, HF hospitalization, change in New York Heart Association (NYHA) class, and change in patient global self-assessment. A reduction in the secondary endpoint of all-cause mortality was also reported. In the RESULT study,^6^ the reduction by RM of the composite endpoint of all-cause death and hospitalization due to cardiovascular reasons was driven by a lower hospitalization rate. Conversely, the REM-HF study,^7^ which evaluated RM using weekly downloads, failed to show a decrease of mortality or hospitalization for cardiovascular reason among patients with HF and CIED.

No study had addressed RM specifically in patients with CRT devices. The ECOST-CRT study was designed to gain a better understanding of the benefits of RM in HF patients with a CIED, and focused on a specific population of HF patients with cardiac resynchronization therapy with (CRT-D) or without defibrillator [cardiac resynchronization therapy pacemaker (CRT-P)]. We conducted this multicentre, randomized, controlled trial to determine whether a ‘comprehensive RM scheme’ with an accurate management plan based on remotely assessed signs and symptoms of HF and notification about daily monitored parameters related to HF would reduce the combined endpoint of death from any cause and hospitalization for worsening HF (WHF), as compared to ‘standard’ RM of technical parameters and ventricular arrhythmias.

## Methods

### Study design

Eligible patients were 18 years of age or older, had received a *de novo* CRT-P or CRT-D device in accordance with the European guidelines,^8^ and had RM activated. Device replacement, lead model under advisory, non-functional lead, participation in another research, or evaluation programme concerning the follow-up (FU) of HF was criteria for exclusion.

**Figure 1:**
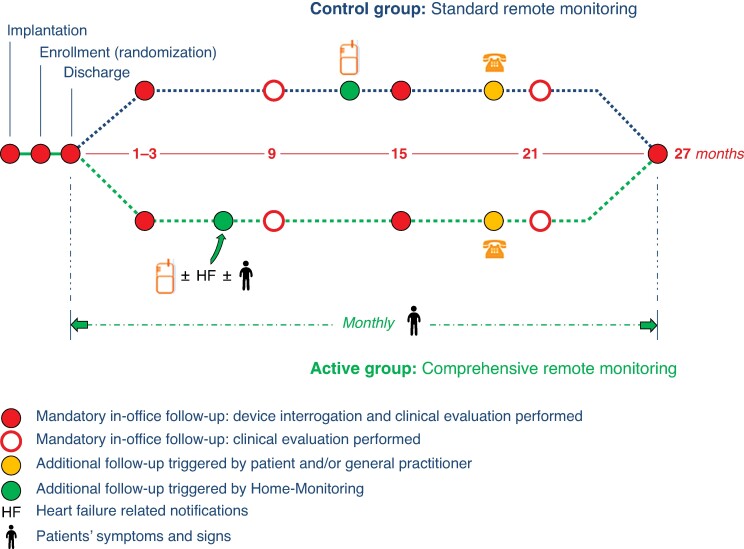
Design of the ECOST-CRT study.

After obtaining written informed consent, eligible patients were randomly assigned in a 1:1 ratio to the control or comprehensive RM (active) group before hospital discharge (*Figure 1*). The trial was conducted in 45 study sites across France (see Supplementary material online), had obtained approval by the ethics committee, and complied with the Declaration of Helsinki.

The RM system (Home-Monitoring®, Biotronik SE and Co. KG, Berlin, Germany) transmits every day technical and medical data and presents them on a secure internet site accessible only to the treating physician. The user can select event types for which he or she wants to be notified.

**Figure 2:**
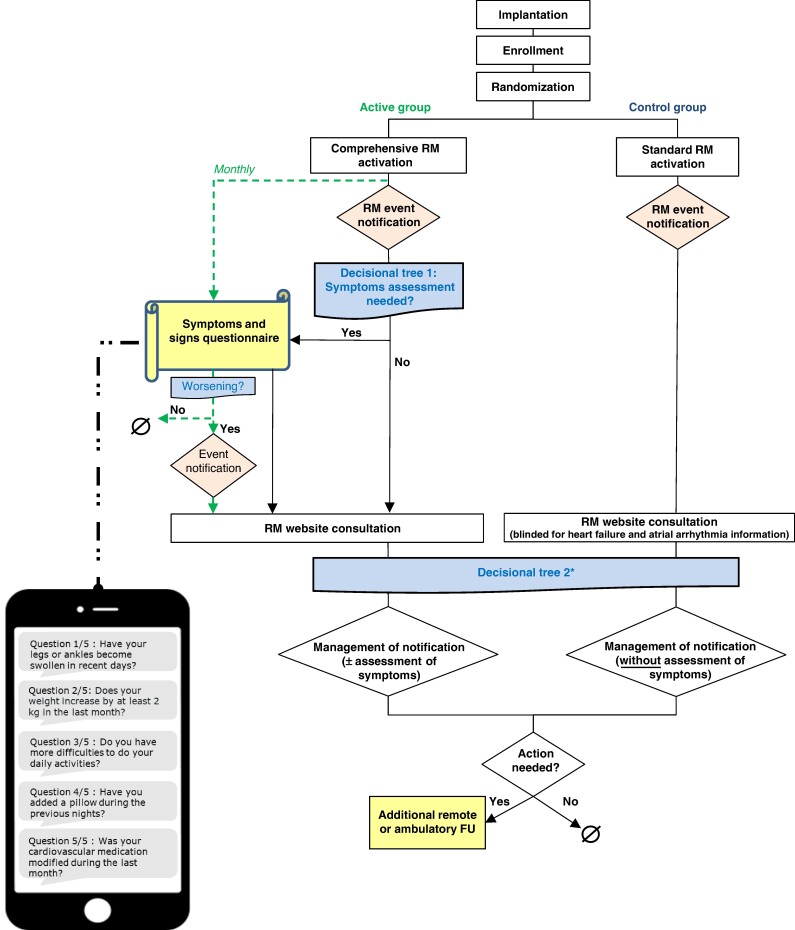
Workflow for remote monitoring management.

In both groups, automatic daily RM was activated and technical parameters (battery status, lead pacing thresholds, pacing impedances, sensing amplitudes, shock impedance) and ventricular arrhythmias (detected arrhythmias and delivered therapies) were selected to trigger event notifications. For the duration of the study, any interruption of transmission for more than 7 days was immediately corrected in both groups. In the active group, signs and symptoms of HF obtained from a questionnaire and RM transmitted parameters related to the acute HF status triggered automatic event notifications to the treating physician, in order to achieve an earlier detection of WHF. Questionnaires with five questions about signs, symptoms of HF, and ongoing cardiovascular treatment (*Figure 2*) were automatically sent to patients on a monthly basis. Remote monitoring parameters related to HF and their threshold for notification were as follows: biventricular (BiV) pacing rate < 95%, mean number of PVCs per hour above 250, mean ventricular rate at rest > 70 beats per minute (bpm), and atrial arrhythmias (atrial burden above 4 h per day and long atrial episode above 6 h). The monthly questionnaires were complemented by equal questionnaires that were triggered if the heart rate at rest was above 70 bpm or if mean PVC per hour was above 250. An automatized Short Message Service (SMS) webapp with simple user-friendly interface was used to send questionnaires and store the answers in the electronic case report form. In case of signs or symptoms of HF or if HF-related parameters crossed pre-defined thresholds, the investigator was notified and decided, based on the questionnaire and on all available RM data, whether or not an additional face-to-face FU was required. If the patient did not respond to the questionnaire, an investigational site member contacted the patient by phone to fill the questionnaire.

In the control group, no questionnaires were sent. All RM parameters were available for their physicians in the RM platform but HF-related parameters did not trigger notifications nor study procedures.

The study protocol did not include regular checks of RM data at calendar intervals. If notifications were received, they had to be managed on the day they were received on the RM platform, or the following working day. The management was performed according to a procedural guide and decisional trees, which may lead to additional face to face or remote FU. The workflow (*Figure 2*) details the actions to be taken after a RM event notification according to patient’s study group. All medical treatments including the decision to hospitalize a patient because of a RM notification were left to the investigators’ discretion.

In addition to RM, standard care was provided to the patient according to guidelines.^8,9^ Scheduled in-office visits with clinical assessment were mandatory at 1–3, 9, 15, 21, and 27 months FU with a device interrogation mandatory at Months 1–3, 15, and 27 in both groups. A quality of life (QoL) questionnaire based on Minnesota Living with Heart Failure (MLHF)^10^ was completed at each scheduled FU. The ECOST-CRT trial is registered at ClinicalTrials.gov (NCT03012490). The study was approved by appropriate competent authorities, and all sites obtained approval from the ethics committees. All patients provided written informed consent.

### Study objectives

The main objective was to demonstrate the efficacy and the safety of the described comprehensive RM scheme of HF patients implanted with a CRT device compared to standard RM. The primary endpoint was a composite of death from any cause and WHF hospitalizations, and was compared between the study groups. A clinical events committee, blinded to patients’ group assignment, adjudicated all deaths and hospitalizations. The clinically relevant secondary outcomes were as follows: deaths (all, HF related, cardiac, or non-cardiac), hospitalization for WHF, cardiovascular-related serious adverse events (SAE) other than hospitalization for WHF, atrial burden, improvement of patient’s clinical status [evolution of left ventricular ejection fraction (LVEF) and NYHA class], QoL based on MLHF questionnaire (scores range from 0 to 105), with score under 24 considered as good, between 24 and 45 as moderate, and over 45 as poor, and analysis of RM notifications related to trends including BiV pacing.

### Premature study termination

Because of the COVID-19 pandemic, many hospitals were forced to adapt their CIEDs’ FU scheme. Patients not seen at face-to-face visits required careful analysis of RM data irrespective of their group assignment, and the protocol required scheme could not be upheld. Therefore, the steering committee and sponsor agreed to terminate the study on 4 May 2020. Patients still followed in the study at that date exited the study prematurely. The full range of available CIEDs’ notifications was activated for the patients assigned to the control group. Patients in neither group received any further Signs and symptoms questionnaires.

### Statistical methods

To detect a 36% reduction in the hazard of a primary outcome with 80% power, we estimated that 277 patients per group were required (a total of 156 first primary-outcome events). Considering a dropout rate of 15%, we included 652 patients (326 per group). We based our estimate of expected event rates on results of studies^4,11–19^ that observed death, hospitalizations for HF, or a similar primary criteria proportion. The main analysis for primary and secondary clinical outcomes was performed on an intention-to-treat (ITT) basis. A descriptive analysis of baseline clinical characteristics was first performed. The normal distribution of variables was verified, using graphical methods such as histograms, and on Shapiro–Wilk test. Comparisons were performed by using a Student’s *t*-test, after confirmation of the equality of variances by Levene’s test or the Mann–Whitney test where appropriate for continuous variables. For analyses of categorical data, χ^2^ tests were used unless there were observed cell counts of <5. In those cases, Fisher’s exact tests were used. For the primary hypothesis and any other inferential analyses, the result of a two-sided statistical test with a *P*-value of less than 5% or a one-sided statistical test with a *P*-value of less than 2.5% was considered statistically significant.

The occurrence of the primary endpoint was evaluated during patient’s FU period to calculate the time-to-event. We used the Kaplan–Meier method to graphically represent their occurrence over time. The patient’s survival time until the occurrence of the studied event was individual. Patients lost to FU were considered as censored. The incidence rate of death or first hospitalization for WHF was compared between both groups using the log-rank test. The hazard ratio and the 95% confidence interval were computed using a Cox proportional hazards model. For all relevant parameters, 95% confidence intervals were given, and for all tests, a significance level of *P* < 0.05 was considered statistically significant. The SAS version 9.4 (SAS Institute Inc., Cary, NC, USA) statistical software was used for the analyses.

## Results

### Recruitment, patient characteristics, and follow-up

Between February 2017 and January 2020, 652 patients were prospectively enrolled (323 in the control and 329 in the active group). Patients’ baseline characteristics were similar in both groups (*Table 1*).

**Table 1:** Characteristics of patients at baseline

	Control group	Active group	*P*-value
	*n* = 323	*n* = 329	
Men	234 (72.5)	245 (74.5)	0.56
Age	70.4 ± 10.5	70.3 ± 10.0	0.89
BMI	28.5 ± 9.5	27.8 ± 5.0	0.71
NYHA			
I	8 (2.5)	8 (2.4)	0.83
II	142 (44.0)	163 (49.6)	0.18
III	135 (41.8)	119 (36.2)	0.16
IV	16 (4.9)	11 (3.3)	0.40
Missing	22 (6.8)	28 (8.5)	0.50
LVEF mean, in %	29.6 ± 7.0	28.6 ± 8.1	0.08
LVEF			0.58
≤35%	288 (89.2)	289 (87.8)	
>35%	34 (10.5)	37 (11.3)	
QRS width in ms	156.3 ± 29.8	154.9 ± 30.2	0.50
*De novo* implantation	239 (74.0)	244 (73.9)	0.94
Upgrade PM	44 (13.6)	46 (13.9)	0.99
Upgrade ICD	40 (12.4)	40 (12.1)	0.99
CRT-P/CRT-D	112 (34.7)/211 (65.3)	98 (29.7)/231 (70.2)	0.17
Ischaemic cardiopathy	145 (44.9)	157 (47.7)	0.48
Dilated cardiopathy	152 (47.1)	138 (42.0)	0.19
Other cardiopathy	84 (26.0)	67 (20.4)	0.09
History of AT/AF	37 (11.5)/161 (49.8)	39 (11.8)/153 (46.5)	0.67/0.64
History of VT/VF	45 (13.9)/3 (1)	42 (12.8)/7 (2)	0.59/0.19
History of LBBB	204 (63.2)	220 (66.9)	0.32
Hypertension	190 (58.8)	198 (60.2)	0.72
Diabetes	110 (34.1)	95 (28.9)	0.15
Dyslipidaemia	133 (41.2)	139 (42.2)	0.78
Hospitalization for WHF in the past year	118 (36.5)	132 (40.1)	0.64
Serum creatinine	115.7 ± 70.4	109.8 ± 42.7	0.76
Beta blockers	276 (85.4%)	284 (86.3%)	0.83
Diuretics	245 (75.9%)	249 (75.7%)	0.97
Lipid-lowering agents	176 (54.5%)	195 (59.3%)	0.25
Anticoagulants	180 (55.7%)	176 (53.5%)	0.62
ACE inhibitors	155 (48.0%)	154 (46.8%)	0.82
Antiplatelets	136 (42.1%)	147 (44.7%)	0.56
Aldosterone blocker	132 (40.9%)	158 (48.0%)	0.08
ARNi	88 (27.2%)	93 (28.3%)	0.84

Data are mean ± SD or *n* (%). There were no significant between-group differences at baseline.

AF, atrial fibrillation; ARNi, angiotensin receptor neprilysin inhibitor; AT, atrial tachycardia; AV, atrioventricular; BMI, body mass index; CRT-D, cardiac resynchronization therapy defibrillator; CRT-P, cardiac resynchronization therapy pacemaker; ICD, implantable cardioverter-defibrillator; LBBB, left bundle branch block; PM, pacemaker; VF, ventricular fibrillation; VT, ventricular tachycardia.

At the time of premature study termination, the planned number of patients had been enrolled, but 364 patients (56%) exited the study prematurely. The mean FU still lasted 18 ± 8 months (median 19, inter-quartile range 11–26 months). Other reasons for premature terminations were: patient death (*n* = 61), lost of FU (*n* = 13), device explantation or cardiac transplantation (*n* = 12), withdrawal of patient consent to study participation (*n* = 5), and patient not compliant to protocol (*n* = 1).

### Clinical outcomes

**Figure 3:**
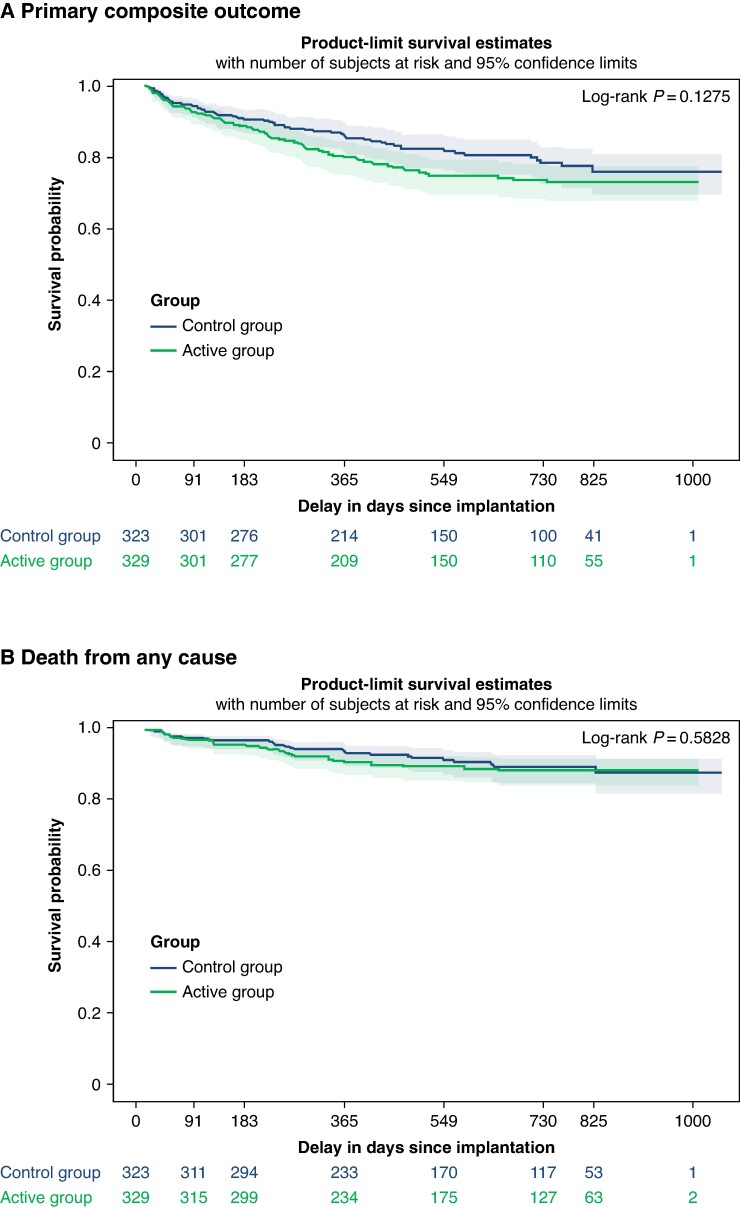
Kaplan–Meier curve for the primary outcomes events in the ITT population. Panel *A* shows the Kaplan–Meier curves for the primary composite outcome of death from any cause or hospitalization for heart failure. Panel *B* shows the Kaplan–Meier curves for death from any cause.

The primary outcome, death from any cause or hospitalization for WHF, was confirmed in 77 of 329 patients (23.3%) in the active group vs. 59 of 323 patients (18.3%) in the control group, without statistically significant difference (log-rank test *P* = 0.13, *Figure 3A* and *Table 2*). No difference was observed between the CRT-P and CRT-D populations nor according to NYHA class.

**Table 2:** Cardiovascular outcomes

Outcome	Control group *n* = 323		Active group *n* = 329		Log-rank *P*-value
	*n*	%	*n*	%	
Primary endpoint					
Hospitalization for WHF^a,b^	41	12.7	52	15.8	–
Death for any cause^b^	18	5.6	25	7.6	–
**Total primary endpoint**	**59**	**18**.**3**	**77**	**23**.**3**	**0**.**13**
Deaths					
Non-cardiac deaths	11	3.4	12	3.6	0.89
Heart failure deaths	8	2.5	11	3.3	0.53
Other cardiovascular deaths	2	0.6	3	0.9	0.66
Unknown cause of death	7	2.2	7	2.1	0.97
Total deaths	28	8.7	33	10.0	0.58
Number of patients with at least one other cardiac SAE	33	10.2	41	12.5	0.61

Bold values correspond to the primary end point of the study.

^a^Hospitalization due to WHF was defined as hospitalization with the occurrence of signs (pulmonary oedema, fluid inflation, cardiogenic shock, or other evidence of WHF) and the need for medication for HF.

^b^Primary endpoint: only the first event within the composite endpoint is considered.

At study closure, 33 (10.0%) patients had died in the active group vs. 28 (8.7%) in the control group (*P* = 0.65) (*Figure 3B*).

Among the study’s secondary objectives, the only statistically significant difference between the study groups was in QoL based on the MLHF questionnaire: the proportion of patients with an improved QoL between baseline and 27 months FU was better in the active group (78% vs. 61%; *P* = 0.03), as was the proportion of patients with good QoL at 27 months FU (74.4% vs. 51.9%, *P* < 0.01).

**Figure 4:**
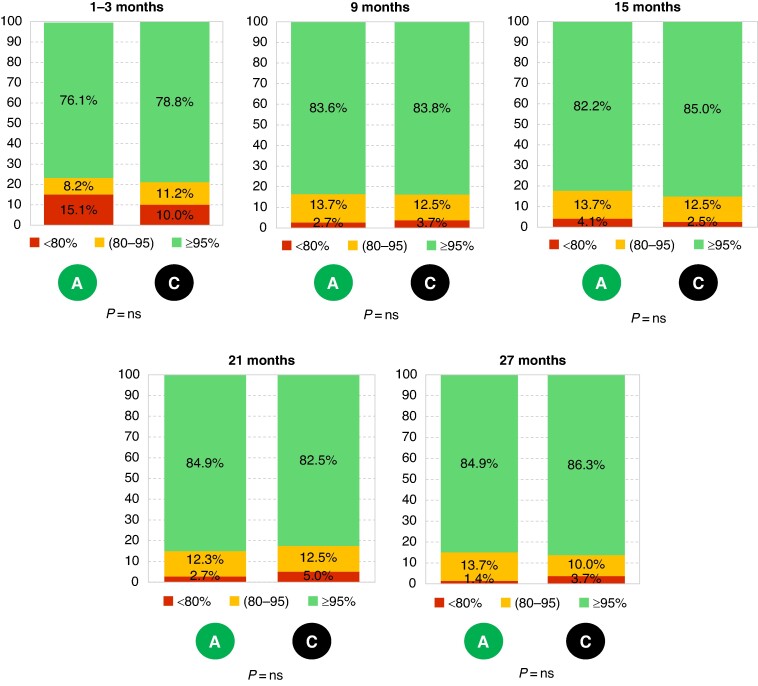
Percentage of BiV pacing in class over the study per group. The percentage of BiV pacing daily transmitted by RM was evaluated at each time in the sub-population of patients who had a complete 27-month FU (73 active patients and 80 control patients).

The percentage of BiV pacing daily transmitted by RM (*Figure 4*) in the sub-population of patients who had a complete 27-month FU (73 active patients and 80 control patients) showed a slightly improvement over time in both groups, without significant difference between them. A total of 84.9% and 86.3% of patients had a percentage of BiV pacing ≥ 95% at 27 months FU in active and control groups, respectively, and 98.6% and 96.3% of patients had a percentage of BiV pacing ≥ 80% at 27 months FU.

The success of daily RM transmissions was 93.0 ± 12.3%.

The number of regular in-office FU at each scheduled FU was similar between both groups (*Table 3*). In addition to these regular in-office FU, there was a total of 559 additional in-office FU (244 in the control group and 315 in the active group). The mean number of additional in-office FU per patient was 0.7 ± 1.2 and 1.0 ± 1.3, respectively, *P* = 0.03. The BiV notifications led to 32 additional follow-ups for 28 patients, and the notifications related to atrial arrhythmias led to 9 additional follow-ups for 9 patients.

**Table 3:** Number of regular in-office follow-ups according to protocol

	Control group	Active group	*P*-value
	*n* = 323	*n* = 329	
1–3 months	298 (92.3%)	305 (92.7%)	0.95
9 months	249 (77.1%)	265 (80.5%)	0.32
15 months	201 (62.2%)	198 (60.2%)	0.65
21 months	140 (43.3%)	155 (47.1%)	0.37
27 months	99 (30.7%)	100 (30.4%)	0.99

### Signs and symptoms patient’s questionnaires

A total of 7662 SMS questionnaires were sent during the study (*Table 4*). In 595 cases (7.8%), the patient did not reply. Five patients (1.5%) did not answer any questionnaire. The replies to 1934 questionnaires (25.2%) contained at least one positive answer. This percentage was similar for monthly (25.4%) and RM event triggered (24.8%) questionnaires. Only 31 questionnaires caused a FU or hospitalization. Of them, 8 (25.8%) were triggered by a RM event notification and led to an in-office FU (6) or hospitalization (2). The remaining 23 (74.2%) questionnaires were among those triggered monthly, 16 (69.6%) leading to an in-office FU and 7 (30.4%) leading to a hospitalization. The 9 hospitalizations resulting from positive questionnaires were related to WHF. For the 22 in-office FU, an intervention was taken for 14 of them [change in cardiovascular treatment (9); change in device programming (4); external shock (1)].

**Table 4:** Type of triggering of the questionnaires SMS related to heart failure

	Patients (*n* = 328)^a^		SMS (*n* = 7 662)		Number SMS per patient (23.4 ± 11.8)
	*n*	%	*n*	%	Mean ± SD
SMS sent monthly	328	100.0	5827	76.0	17.8 ± 7.9
SMS sent after RM event notification	241	73.5	1835	24.0	7.6 ± 8.2
SMS with at least one positive answer	285	86.9	1934	25.2	6.8 ± 6.5
SMS sent monthly	273	83.2	1478	25.4	5.4 ± 4.9
SMS sent after RM event notification	145	44.2	456	24.8	3.1 ± 3.3
SMS with at least one positive answer and for which an action has been triggered (in-office FU or hospitalization)	25	7.6	31	0.4	1.2 ± 0.7

^a^328 patients of the active group received at least one SMS (99.7%). One patient withdrew his consent the day of the signature, therefore he did not receive any questionnaire.

## Discussion

This study does not support the assumption that a comprehensive RM scheme—with notifications triggered by signs and symptoms of HF and by deviations in device-derived heart failure-specific RM data—leads to a lower incidence of a composite of death from any cause or hospitalizations for WHF, when added to notifications about ventricular arrhythmias and technical parameters.

Several factors may account for these results. Telemedicine studies that have shown positive results over the past decade included patients at high risk of decompensation, mainly in the vulnerable period after hospital admission due to HF decompensation.^4,20^ Despite a cohort of patients with reduce LVEF, only 38% of the ECOST-CRT patients had been admitted for WHF in the previous year. Inclusion criteria did not specify a minimum NTproBNP value, and patients with stage B HF (those with ventricular dysfunction but no history of HF decompensations) were eligible for inclusion. In addition, it is expected that a percentage of patients in whom a CRT is implanted *de novo* (the inclusion criterion of this study) will be responders, reducing their risk of WHF. This selection of patients including profiles at low risk of decompensation was probably one of the reasons of the low number of clinical events during the study. Moreover, patients in the control group already benefited from very close monitoring including conventional RM data with notifications of technical events and ventricular arrhythmia. This may also explain the relatively low rate of clinical events observed in the control group compared with that observed in a similar patient population, such as that in the CARE HF and RAFT studies.^12,14^ Furthermore, the RM parameters related to HF that were available in our study may not have been specific enough to the state of congestion, and some parameters may not be worth monitoring daily, especially since the notification threshold is demanding. This is most likely the case for the CRT rate: the 95% cut-off value for triggering an event notification was probably too high to be useful in triggering an intervention in patients, especially since the CRT rate remained very high in the two groups of patients. As for sub-clinical AF, recent data from the NOAH-AFNET 6 and ARTESIA studies^21,22^ have put into perspective the value of screening for the initiation of anticoagulant therapy, with no impact on mortality. Remote monitoring of atrial fibrillation may not reduce the risk of WHF either. As stated in the 2023 expert consensus statement, patients who have underlying HF should necessitate specific RM of their device for signs of HF decompensation.^2^ This is all the more true in the case of resynchronization. But it has never been firmly proven that, for the specific population of resynchronized patients, RM of BiV pacing and/or atrial arrhythmias improves patient prognosis. Remote monitoring of these parameters is a class 2a recommendation with a level of evidence B-R. The results of the ECOST-CRT study do not tend to provide arguments that would raise the level of evidence for RM of these parameters. Also, the potential benefit of RM of symptoms was probably minimized by the frequency of face-to-face FUs in the control group in line with the HF guidelines,^8,9^ which gave patients the opportunity to report their symptoms without too much delay. Moreover, even in the absence of a RM event notification, patients whose condition and therapies had not stabilized were in fact, regardless of the group to which they belonged, reviewed at short notice in scheduled face-to-face consultations in order to adapt their background medication or diuretic treatment. On the other hand, it is plausible that comprehensive RM could further safely reduce the number of useful face-to-face FUs in stabilized patients.

Our study employed a simple to use remote questionnaire. Three results are worth considering. First, patient compliance with the questionnaires was good, as shown by the response rate. Secondly, responses to the questionnaires triggered only a small number (0.4%) of consultations or hospitalizations, but a significant number of phone calls (10%). Maybe investigators or study nurses gave advice during those phone calls, even if no face-to-face visit was arranged. Thirdly, the patients reported a symptom in a similar share of questionnaires that were sent monthly or after RM notifications. This suggests that the HF-related RM parameters used failed to identify symptomatic periods, most likely due to the lower specificity of these parameters for monitoring clinical congestion compared to markers such as chest impedance or pulmonary pressure.

Despite the lack of impact on the incidence of the primary endpoint, the QoL of patients in whom comprehensive RM was carried out significantly improved. This could be explained by the fact that patients have a positive feeling of being closely monitored through the monthly symptom questionnaire and subsequent telephone contacts. But this result must be interpreted with caution, given the small proportion of patients for whom data were available at 27 months. By the way, in the Danish Acquire-ICD study, a web-based intervention had no impact on ICD acceptance and mental health in first-time ICD patients.^23^ In any case, our data suggest that the monthly questionnaire was not a burden on patients. It could be a useful tool for spacing out face-to-face visits for patients with stabilized HF, for example, by associating it with RM of a HF score combining physiological parameters recorded by the CIED.^24^

### Limitations

This trial has several limitations. Owing to the COVID-19 pandemic and the premature termination of the trial, some patients were not followed for the required 27 months. The resulting reduction in number of expected events had an impact on the statistical power of the study. But the decision to provide full RM for all patients during pandemic was benevolent. It would likely have been shared by other experts in view of the data reported by the EHRA physician recent survey that highlighted the impact of COVID-19 on the increased use of RM of CIEDs.^25^

Further, the ECOST-CRT study did not use certain other parameters that are undoubtedly important in the context of HF RM, such as weight values measured several times a week, biological markers, or filling pressures.

## Conclusion

The randomized ECOST-CRT study does not support the notion that comprehensive RM, when compared to standard RM, in HF patients with CRT improves the clinical outcome of all-cause mortality or WHF hospitalizations.

Regular questionnaires related to the patients’ symptoms may improve the patients QoL, but this should be confirmed in further studies. Due to early study termination, the study was underpowered, and the analysis of the primary and secondary endpoints should be interpreted with caution.

## Supplementary Material

euae233_Supplementary_Data

## Data Availability

The data underlying this article will be shared on reasonable request to the corresponding author.
