# Supplementary material for: Comprehensive vs. standard remote monitoring of cardiac resynchronization devices in heart failure patients: results of the ECOST-CRT study
Source: Europace. 2024 Oct 14;26(10):euae233. doi: 10.1093/europace/euae233 (PMC11472153; doi:10.1093/europace/euae233)
Supplement: euae233_Supplementary_Data [file euae233_supplementary_data.docx]

**Supplementary information**

The following investigators and institutions participated in the ECOST-CRT study:

| Site | City | Principal Investigator |
| --- | --- | --- |
| CHU LILLE | LILLE | Laurence GUEDON-MOREAU |
| CHU POITIERS | POITIERS | Bruno DEGAND |
| CH SAINT PHILIBERT | LOMME | Yves GUYOMAR |
| CHU BREST | BREST | Jacques MANSOURATI |
| CHU TOURS | CHAMBRAY-LES-TOURS | Dominique BABUTY |
| CLINIQUE DU MILLENAIRE | MONTPELLIER | Maxime PONS |
| CHU LIMOGES | LIMOGES | Benoit GUY-MOYAT |
| SCAPP TIMONE | MARSEILLE | Jean-Claude DEHARO |
| GROUPE CONFLUENT NANTES | NANTES | Daniel GRAS |
| APHP PITIE SALPETRIERE | PARIS | Caroline HIMBERT |
| CHU RENNES | RENNES | Christophe LECLERCQ |
| CHU MONTPELLIER | MONTPELLIER | Jean-Luc PASQUIE |
| CHU CLERMONT-FERRAND | CLERMONT-FERRAND | Romain ESCHALIER |
| CHU STRASBOURG | STRASBOURG | Halim MARZAK |
| CHR METZ-THIONVILLE | METZ | Michel BOURSIER |
| CH AUXERRE | AUXERRE | François JOURDA |
| CHU ROUEN | ROUEN | Frédéric ANSELME |
| HÔPITAUX DE CHARTRES | LE COUDRAY | Hervé GORKA |
| CHD VENDEE | LA ROCHE SUR YON | Olivier BILLON |
| CHU CÔTE DE NACRE | CAEN | Laure CHAMP-RIGOT |
| HP JACQUES CARTIER | MASSY | Mina AIT SAID |
| CH AIX | AIX-EN-PROVENCE | Jérôme TAIEB |
| CHU BESANCON | BESANCON | Marc BADOZ |
| CH BAYONNE | BAYONNE | Julien LABORDERIE |
| CH MARNE-LA-VALLEE | JOSSIGNY | Mohamed BELHAMECHE |
| CHU BORDEAUX | PESSAC | Sylvain PLOUX |
| CH PAU | PAU | Maxime de GUILLEBON |
| CH ANNECY GENEVOIS | ANNECY | Antoine DOMPNIER |
| CLINIQUE PASTEUR TOULOUSE | TOULOUSE | Serge BOVEDA |
| CLINIQUE SAINT MARTIN | CAEN | Sophie GOMES-FERREIRA |
| POLE SANTE ORELIANCE | ORLEANS | Cédric GIRAUDEAU |
| SCAPP NORD | MARSEILLE | Michael PEYROL |
| CHU NIMES | NIMES | Pierre WINUM |
| CH SAINT JOSEPH SAINT LUC | LYON | Benjamin GAL |
| CHU NANCY | VANDOEUVRE-LES-NANCY | Hugues BLANGY |
| CH VILLEFRANCHE SUR SAÔNE | GLEIZE | Olivier LE VAVASSEUR |
| CHU TOULOUSE | TOULOUSE | Alexandre DUPARC |
| CH VALENCIENNES | VALENCIENNES | Laura FORELLE |
| CLINIQUE SAINT JOSEPH | TRELAZE | Albin BEHAGHEL |
| HÔPITAL NORD FRANCHE COMTE | BELFORT | Renaud FOUCHE |
| CHU DU BOCAGE | DIJON | Gabriel LAURENT |
| CH MOULINS | MOULINS | Hassan BARAKE |
| CL SAINT AUGUSTIN | BORDEAUX | Sylvain REUTER |
| CH PERPIGNAN | PERPIGNAN | Pierre SULTAN |
| CHU SAINT-ETIENNE | SAINT-ETIENNE | Antoine DA COSTA |
